# Risk factors for recurrence after initial transurethral resection of bladder tumor in patients with non–muscle-invasive bladder cancer

**DOI:** 10.3389/fonc.2026.1861461

**Published:** 2026-10-01

**Authors:** Fang Yuan, Xiong Li, Jing Zhang, Jian-Yong Gao

**Affiliations:** 1 Department of Urology, The Affiliated Hospital of Southwest Medical University, Luzhou, Sichuan, China; 2 Department of Urology, Panzhihua Central Hospital, Panzhihua, Sichuan, China

**Keywords:** carcinoma in situ, intravesical therapy, neutrophil-to-lymphocyte ratio, non–muscle-invasive bladder cancer, recurrence, transurethral resection of bladder tumor

## Abstract

**Background:**

Recurrence remains common after initial transurethral resection of bladder tumor (TURBT) for non–muscle-invasive bladder cancer (NMIBC). This study aimed to identify routinely available clinicopathological, treatment-related, and inflammatory factors associated with recurrence and to characterize recurrence-free survival (RFS).

**Methods:**

This retrospective cohort included patients with pathologically confirmed Ta, T1, or primary carcinoma *in situ* (CIS) who underwent initial TURBT between January 2017 and December 2023. Multivariable logistic regression was used as the primary analysis to evaluate factors associated with documented recurrence during follow-up. Cox proportional hazards regression was performed as a complementary analysis incorporating recurrence timing and censoring. Kaplan–Meier analysis, sensitivity analyses, restricted cubic spline analysis, time-dependent receiver operating characteristic analysis, and bootstrap-corrected model evaluation were also performed.

**Results:**

Among 605 patients, 237 (39.2%) experienced recurrence during a median follow-up of 34.0 months. Multiple tumors were associated with higher recurrence odds and hazard (adjusted odds ratio [OR], 1.88; 95% confidence interval [CI], 1.27–2.78; adjusted hazard ratio [HR], 1.62; 95% CI, 1.25–2.10). Higher recurrence odds and hazards were also associated with increasing tumor diameter, T1 or primary CIS, high-grade pathology, concomitant CIS, and higher baseline neutrophil-to-lymphocyte ratio. Maintenance intravesical therapy was inversely associated with recurrence (adjusted OR, 0.49; 95% CI, 0.34–0.72; adjusted HR, 0.62; 95% CI, 0.47–0.81). Estimated RFS rates were 81.5%, 63.8%, and 54.1% at 1, 3, and 5 years, respectively. The bootstrap-corrected area under the curve was 0.769 for the logistic model, and the bootstrap-corrected Harrell’s C-index was 0.739 for the Cox model.

**Conclusions:**

Recurrence after initial TURBT was associated with greater tumor burden, adverse pathological characteristics, concomitant CIS, and higher baseline neutrophil-to-lymphocyte ratio. Maintenance intravesical therapy showed an inverse observational association with recurrence. These routinely available factors may support postoperative risk stratification and risk-adapted surveillance.

## 1 Introduction

Bladder cancer remains a major urologic malignancy, with approximately three quarters of newly diagnosed cases classified as non–muscle-invasive bladder cancer (NMIBC) (1). Although transurethral resection of bladder tumor (TURBT) is the standard initial treatment, NMIBC is characterized by substantial biological heterogeneity and a persistent risk of intravesical recurrence (2). Recurrent disease often requires repeated endoscopic procedures, additional intravesical therapy, and prolonged cystoscopic surveillance, making recurrence a major contributor to the clinical and economic burden of NMIBC (3, 4).

Current risk assessment relies primarily on established clinicopathological factors, including tumor number, size, stage, grade, and concomitant carcinoma *in situ* (CIS) (5). However, conventional models provide only moderate discrimination, particularly in patients receiving contemporary intravesical therapy. Accordingly, increasing attention has been directed toward adjunctive predictors available in routine practice, including systemic inflammatory markers, anthropometric and lifestyle-related factors, and laboratory parameters (6, 7). Recent studies have reported associations between inflammatory indices and postoperative outcomes, while prospective cohorts have evaluated cancer-prevention behaviors, body mass index, abdominal adiposity, and physical activity (8–10). Urine-based molecular biomarkers have also been explored as complements to cystoscopic surveillance. In a prospective active-surveillance cohort of patients with recurrent, predominantly low-grade NMIBC, serially negative Xpert Bladder Cancer Monitor results were associated with the potential omission of some scheduled cystoscopies (11). Collectively, these findings support a more integrated approach incorporating tumor-, host-, treatment-, and biomarker-related information.

Recent studies have sought to improve postoperative risk stratification using nomograms, externally validated prediction models, and artificial intelligence–based approaches (12–14). These developments reflect growing interest in more individualized recurrence assessment to support risk-adapted surveillance, treatment planning, and patient counseling. However, some advanced models depend on specialized imaging, digital pathology, or molecular platforms that are not routinely available across institutions, while the prognostic contribution of readily obtainable clinical variables remains incompletely characterized (15, 16). In particular, relatively few studies have jointly evaluated clinicopathological characteristics, postoperative treatment exposure, and systemic inflammatory markers while accounting for variation in follow-up duration and the classification of early postoperative lesions (17, 18). Therefore, the present study investigated factors associated with documented recurrence and recurrence-free survival after initial TURBT in patients with NMIBC, with particular emphasis on routinely available variables that may contribute to practical postoperative risk stratification.

## 2 Methods

### 2.1 Study design

This retrospective cohort study reviewed patients who underwent initial TURBT for suspected NMIBC at our institution between January 2017 and December 2023. Patients were eligible if postoperative pathological examination confirmed Ta, T1, or primary CIS, the initial TURBT was evaluable, and sufficient clinicopathological and follow-up data were available for recurrence assessment. Patients were excluded if postoperative pathological examination confirmed muscle-invasive bladder cancer; if they had upper urinary tract urothelial carcinoma, distant metastasis, a previous history of bladder cancer or prior bladder-directed treatment, or a concurrent active malignancy; or if they had undergone a grossly incomplete or aborted resection that precluded reliable baseline assessment. Additional exclusion criteria were perioperative death, severe hematologic, hepatic, or renal dysfunction, active severe infection, autoimmune disease, absence of a definitive pathological diagnosis, or missing key baseline or follow-up data. Informed consent was obtained from all participants and/or their legal guardians. All procedures were conducted in accordance with the Declaration of Helsinki and relevant ethical guidelines and regulations. All data were anonymized before analysis to protect participant confidentiality.

### 2.2 Definition and adjudication of recurrence

The primary outcome was intravesical tumor recurrence after initial TURBT. Recurrence was defined as a newly detected bladder tumor during follow-up, identified by cystoscopy and supported, when available, by imaging and/or histopathological findings. Recurrence-free survival was calculated from the date of initial TURBT to the first documented recurrence. Patients without documented recurrence were censored at death, the last available clinical assessment, or the administrative end of follow-up, whichever occurred first. Lesions detected within 3 months after TURBT were retrospectively adjudicated using operative records, documented resection completeness, lesion location, repeat TURBT findings, and subsequent pathological results. Lesions considered to represent persistence of the original tumor were classified as residual disease, whereas lesions judged to represent newly developed tumors were classified as recurrence. Lesions of uncertain classification were not counted as recurrence events. A sensitivity analysis excluded all patients with lesions detected within 3 months. Consistent with the study objective, patients were classified according to whether recurrence was documented during the observed follow-up for the primary association analysis. Because follow-up duration varied, Kaplan–Meier estimation of recurrence-free survival and Cox proportional hazards regression were additionally performed as complementary time-to-event analyses incorporating recurrence timing and censoring. A sensitivity analysis excluding non-recurrent patients followed for less than 12 months was also conducted.

### 2.3 Surgical and postoperative management

All patients underwent initial TURBT at our institution, with complete removal of visible tumors and resection of the tumor base attempted whenever technically feasible. Additional mucosal or detrusor muscle specimens were obtained when clinically indicated. Tumor number, size, and location were extracted from operative records, whereas pathological stage, grade, primary CIS, and concomitant CIS were determined from institutional pathology reports. Complete resection was defined as explicit operative documentation that all visible tumors had been removed. Cases with uncertain or undocumented resection completeness were coded separately from grossly incomplete or aborted procedures that met the exclusion criteria. The presence of detrusor muscle in the initial TURBT pathology specimen, the resection technique (en bloc or conventional), and the use of enhanced imaging were retrospectively extracted from pathology and operative records when available. Postoperative intravesical treatment was retrospectively classified as immediate instillation, induction therapy, or maintenance therapy. Regimens were categorized as BCG-based or chemotherapy-based and included pirarubicin, epirubicin, mitomycin C, and other documented agents. When available, the number of instillations, treatment duration, regimen completion, interruption or discontinuation, and the corresponding reasons were recorded. Treatment selection was determined in routine practice according to clinicopathological characteristics, treatment tolerance, patient preference, and drug availability. Accordingly, treatment-related findings were interpreted as observational associations. Repeat TURBT was performed when clinically indicated, particularly in selected patients with T1 disease or uncertain resection completeness, and relevant procedural and pathological findings were extracted from the medical records.

### 2.4 Data collection and variable definitions

Clinical data were retrospectively extracted from electronic medical records, operative and pathology reports, laboratory databases, intravesical treatment records, and follow-up files using a standardized form. Data were independently reviewed by two investigators, and discrepancies were resolved by referring to the original records. Collected variables included demographic characteristics, comorbidities, presenting urinary symptoms, tumor and pathological features, perioperative characteristics, postoperative intravesical treatment, and preoperative laboratory parameters. Tumor-related variables comprised tumor number, maximum diameter, anatomical location, pathological stage, grade, primary CIS, concomitant CIS, and multifocality. Tumor number was categorized as solitary or multiple. Pathological stage was classified as Ta, T1, or primary CIS; for the main regression analyses, T1 and primary CIS were combined and compared with Ta. Stage-stratified analyses were conducted separately for Ta and T1 disease, while primary CIS was summarized descriptively. Perioperative and treatment-related variables included operative time, documented completeness of resection, obturator nerve reflex, bladder perforation, immediate postoperative instillation, induction therapy, maintenance therapy, and repeat TURBT. Preoperative laboratory values were obtained from the most recent tests performed before TURBT and included hemoglobin, white blood cell, neutrophil, lymphocyte, and platelet counts, serum albumin, and serum creatinine. The neutrophil-to-lymphocyte ratio (NLR) and platelet-to-lymphocyte ratio (PLR) were calculated by dividing the neutrophil and platelet counts, respectively, by the lymphocyte count. The SII was calculated as the platelet count multiplied by the neutrophil count and divided by the lymphocyte count. Because these inflammatory measures share mathematical components, their correlations were assessed, and they were not entered simultaneously into the final multivariable models. NLR was retained as the principal inflammatory marker.

### 2.5 Follow-up

Follow-up information was retrospectively obtained from outpatient and inpatient records, telephone interviews, and the institutional electronic medical record system. Surveillance assessments included cystoscopy, urine cytology, imaging when clinically indicated, and histopathological evaluation when tissue specimens were available. Cystoscopy was generally scheduled every 3 months during the first 2 years after TURBT, every 6 months thereafter, and annually after 5 years, with intervals adjusted according to pathological characteristics and clinical circumstances. Follow-up ended at documented recurrence, death, the last available clinical assessment, or the administrative cutoff of December 2025, whichever occurred first. For non-recurrent patients followed for less than 12 months, the reason for early follow-up termination was retrospectively reviewed. Surveillance intensity was assessed using the time to the first postoperative cystoscopy, the total number of cystoscopies, the number of cystoscopies per patient-year, and the occurrence of delayed or missed surveillance visits.

### 2.6 Statistical analysis

All statistical analyses were performed using IBM SPSS Statistics, version 30.0 (IBM Corp., Armonk, NY, USA). Continuous variables were summarized as the mean ± standard deviation or median with interquartile range and compared using the independent-samples t test or Mann–Whitney U test, as appropriate. Categorical variables were presented as counts and percentages and compared using the Pearson chi-square test or Fisher’s exact test. Univariable and multivariable logistic regression analyses were used as the primary association analyses to identify factors associated with whether recurrence was documented during the observed follow-up, with odds ratios (ORs) and 95% confidence intervals (CIs) reported. Because recurrence was also a time-to-event outcome and follow-up duration varied, Cox proportional hazards regression was performed as a complementary analysis incorporating recurrence timing and censoring, with hazard ratios (HRs) and 95% CIs reported. These approaches addressed related but distinct estimands: logistic regression evaluated overall documented recurrence status during follow-up, whereas Cox regression evaluated recurrence hazard over time. The Cox analysis was used to assess the temporal consistency of the observed associations rather than to replace the prespecified primary analysis. Variables were selected for multivariable modeling based on univariable associations, clinical relevance, potential confounding, and collinearity diagnostics. To reduce mathematical and biological redundancy, the NLR was retained as the principal inflammatory marker, whereas its component cell counts and related composite inflammatory indices were not entered simultaneously. Variables representing presenting symptoms, procedural consequences, or overlapping measures of tumor burden were not retained in the final multivariable models. Spearman correlation coefficients, tolerance values, and variance inflation factors were used to assess correlation and multicollinearity. Recurrence-free survival was estimated using the Kaplan–Meier method and compared using the log-rank test. The proportional hazards assumption was assessed using Schoenfeld residuals. Stage-stratified analyses included formal interaction tests. Sensitivity analyses excluded patients who underwent repeat TURBT, non-recurrent patients followed for less than 12 months, and patients with lesions detected within 3 months after TURBT. Absolute standardized mean differences were used to compare baseline characteristics between non-recurrent patients with shorter and longer follow-up. Restricted cubic spline analysis was used to assess the shape of the association between NLR and recurrence hazard. Time-dependent receiver operating characteristic (ROC) analysis evaluated the discriminatory performance of NLR at 1, 3, and 5 years. Model performance was assessed using the area under the curve (AUC), calibration intercept, calibration slope, Brier score, Harrell’s C-index, and time-dependent AUCs, with optimism correction performed using nonparametric bootstrap resampling. All tests were two-sided, and P < 0.05 was considered statistically significant.

## 3 Results

### 3.1 Patient selection and follow-up outcomes

A total of 656 patients who underwent initial TURBT for suspected NMIBC at our institution between January 2017 and December 2023 were retrospectively screened. After eligibility assessment, 51 patients were excluded: 14 because of missing key clinicopathological data, 11 because of insufficient follow-up information for recurrence assessment, 9 because of a previous history of bladder cancer or prior bladder-directed treatment, 7 because postoperative pathological examination confirmed muscle-invasive bladder cancer, 6 because of concurrent upper urinary tract urothelial carcinoma or another active malignancy, and 4 because of a grossly incomplete or aborted resection that precluded reliable baseline assessment or because no definitive pathological diagnosis was available. Consequently, 605 patients with pathologically confirmed Ta, T1, or primary CIS were included in the final analysis. During follow-up, 237 patients (39.2%) had documented recurrence and 368 (60.8%) had no documented recurrence. The median follow-up duration was 34.0 months (interquartile range [IQR], 22.0–51.0 months). Among patients with recurrence, the median time from initial TURBT to recurrence was 15.0 months (IQR, 8.0–28.0 months). Follow-up status was available for all included patients. Forty-seven patients without documented recurrence had less than 12 months of follow-up, and the reasons for early follow-up termination were subsequently examined in supplementary analyses.

### 3.2 Baseline demographic and clinical characteristics

The mean age of the overall cohort was 65.0 ± 9.1 years, and 455 patients (75.2%) were male. Compared with patients without documented recurrence, those with recurrence were older, had a higher body mass index, and more frequently had a history of smoking, hypertension, diabetes mellitus, or previous urinary system disease. Urinary frequency, urinary urgency, and dysuria were also more frequent among patients with recurrence. No statistically significant between-group differences were observed in sex, alcohol consumption, coronary artery disease, gross hematuria, or other lower urinary tract symptoms (**Table 1**).

**Table 1:** Baseline demographic and clinical characteristics.

Variable	Overall cohort (n = 605)	Recurrence group (n = 237)	Non-recurrence group (n = 368)	Test statistic	P value
Age, years	65.0 ± 9.1	66.8 ± 9.4	63.9 ± 8.7	t = 3.88	<0.001
Male sex, n (%)	455 (75.2)	183 (77.2)	272 (73.9)	χ² = 0.84	0.359
BMI, kg/m²	24.8 ± 3.2	25.3 ± 3.4	24.4 ± 3.1	t = 3.36	0.001
Smoking history, n (%)	255 (42.1)	119 (50.2)	136 (37.0)	χ² = 10.39	0.001
Alcohol consumption history, n (%)	206 (34.0)	86 (36.3)	120 (32.6)	χ² = 0.87	0.351
Hypertension, n (%)	287 (47.4)	126 (53.2)	161 (43.8)	χ² = 5.12	0.024
Diabetes mellitus, n (%)	159 (26.3)	74 (31.2)	85 (23.1)	χ² = 4.91	0.027
Coronary artery disease, n (%)	99 (16.4)	43 (18.1)	56 (15.2)	χ² = 0.90	0.342
Previous urinary system disease history, n (%)	156 (25.8)	75 (31.6)	81 (22.0)	χ² = 6.99	0.008
Gross hematuria, n (%)	419 (69.3)	165 (69.6)	254 (69.0)	χ² = 0.02	0.876
Urinary frequency, n (%)	221 (36.5)	103 (43.5)	118 (32.1)	χ² = 8.07	0.004
Urinary urgency, n (%)	219 (36.2)	101 (42.6)	118 (32.1)	χ² = 6.95	0.008
Dysuria, n (%)	161 (26.6)	74 (31.2)	87 (23.6)	χ² = 4.24	0.039
Other lower urinary tract symptoms, n (%)	142 (23.5)	59 (24.9)	83 (22.6)	χ² = 0.44	0.507

BMI, body mass index.

### 3.3 Tumor-related, pathological, perioperative, treatment-related, and laboratory characteristics

Multiple tumors were present in 302 patients (49.9%), T1 disease in 257 (42.5%), primary CIS in 51 (8.4%), high-grade disease in 332 (54.9%), and concomitant CIS in 95 (15.7%). Patients with recurrence more frequently had multiple tumors, larger tumors, T1 or primary CIS, high-grade pathology, concomitant CIS, and multifocal disease. They also had a longer operative time and higher frequencies of obturator nerve reflex and repeat TURBT, whereas complete resection, immediate postoperative intravesical instillation, and maintenance intravesical therapy were less frequent.

Regarding laboratory characteristics, the recurrence group had lower mean hemoglobin, lymphocyte, and albumin levels and higher white blood cell, neutrophil, platelet, NLR, PLR, and SII values. Tumor location, bladder perforation, induction intravesical therapy, and serum creatinine did not differ significantly between the two groups (**Table 2**).

**Table 2:** Tumor-related, pathological, perioperative, treatment-related, and laboratory characteristics.

Variable	Overall cohort (n = 605)	Recurrence group (n = 237)	Non-recurrence group (n = 368)	Test statistic	P value
Tumor and pathological characteristics					
Tumor number, n (%)				χ² = 19.77	<0.001
Solitary	303 (50.1)	92 (38.8)	211 (57.3)		
Multiple	302 (49.9)	145 (61.2)	157 (42.7)		
Maximum tumor diameter, cm	2.7 ± 1.1	3.1 ± 1.2	2.4 ± 1.0	t = 7.46	<0.001
Tumor location, n (%)				χ² = 1.42	0.841
Lateral wall	195 (32.2)	74 (31.2)	121 (32.9)		
Posterior wall	150 (24.8)	61 (25.7)	89 (24.2)		
Trigone	90 (14.9)	38 (16.0)	52 (14.1)		
Bladder neck	42 (6.9)	18 (7.6)	24 (6.5)		
Multiple locations	128 (21.2)	46 (19.4)	82 (22.3)		
Pathological stage, n (%)				χ² = 11.50	0.003
Ta	297 (49.1)	96 (40.5)	201 (54.6)		
T1	257 (42.5)	118 (49.8)	139 (37.8)		
Primary CIS	51 (8.4)	23 (9.7)	28 (7.6)		
Pathological grade, n (%)				χ² = 21.87	<0.001
Low-grade	273 (45.1)	79 (33.3)	194 (52.7)		
High-grade	332 (54.9)	158 (66.7)	174 (47.3)		
Concomitant CIS, n (%)	95 (15.7)	52 (21.9)	43 (11.7)	χ² = 11.46	<0.001
Multifocality, n (%)	204 (33.7)	101 (42.6)	103 (28.0)	χ² = 13.80	<0.001
Perioperative characteristics					
Operative time, min	32.8 ± 11.0	34.8 ± 11.6	31.5 ± 10.4	t = 3.56	<0.001
Complete resection, n (%)	556 (91.9)	211 (89.0)	345 (93.8)	χ² = 4.32	0.038
Obturator nerve reflex, n (%)	73 (12.1)	39 (16.5)	34 (9.2)	χ² = 7.08	0.008
Bladder perforation, n (%)	21 (3.5)	11 (4.6)	10 (2.7)	χ² = 1.59	0.207
Repeat TURBT, n (%)	99 (16.4)	49 (20.7)	50 (13.6)	χ² = 5.29	0.021
Treatment-related characteristics					
Immediate postoperative intravesical instillation, n (%)	449 (74.2)	162 (68.4)	287 (78.0)	χ² = 6.99	0.008
Induction intravesical therapy, n (%)	448 (74.0)	168 (70.9)	280 (76.1)	χ² = 2.03	0.154
Maintenance intravesical therapy, n (%)	367 (60.7)	121 (51.1)	246 (66.8)	χ² = 15.07	<0.001
Laboratory parameters					
Hemoglobin, g/L	130.1 ± 16.9	127.4 ± 17.3	131.8 ± 16.5	t = -3.11	0.002
White blood cell count, ×10^9^/L	7.0 ± 2.0	7.3 ± 2.1	6.8 ± 1.9	t = 2.97	0.003
Neutrophil count, ×10^9^/L	4.4 ± 1.6	4.8 ± 1.7	4.2 ± 1.5	t = 4.43	<0.001
Lymphocyte count, ×10^9^/L	1.75 ± 0.51	1.63 ± 0.48	1.82 ± 0.51	t = -4.64	<0.001
Platelet count, ×10^9^/L	240 ± 64	248 ± 67	235 ± 61	t= 2.41	0.016
Albumin, g/L	39.6 ± 4.6	38.6 ± 4.7	40.2 ± 4.4	t = -4.19	<0.001
Creatinine, μmol/L	84.1 ± 20.3	86.1 ± 21.5	82.8 ± 19.4	t = 1.91	0.056
NLR	2.92 ± 1.36	3.35 ± 1.46	2.64 ± 1.21	t = 6.23	<0.001
PLR	152 ± 55	162 ± 58	146 ± 52	t = 3.45	<0.001
SII	708 ± 307	792 ± 318	654 ± 287	t = 5.41	<0.001

CIS, carcinoma *in situ*; NLR, neutrophil-to-lymphocyte ratio; PLR, platelet-to-lymphocyte ratio; SII, systemic immune-inflammation index; TURBT, transurethral resection of bladder tumor.

### 3.4 Univariable logistic regression analysis

In the univariable logistic regression analyses, older age, higher body mass index, smoking history, hypertension, diabetes mellitus, previous urinary system disease, urinary frequency, urinary urgency, and dysuria were associated with higher odds of documented recurrence. Tumor-related factors associated with recurrence included multiple tumors, larger maximum tumor diameter, T1 or primary CIS, high-grade pathology, concomitant CIS, and multifocality. Longer operative time, obturator nerve reflex, and repeat TURBT were also associated with higher recurrence odds, whereas complete resection, immediate postoperative intravesical instillation, and maintenance intravesical therapy were associated with lower recurrence odds. Among laboratory parameters, lower hemoglobin, lymphocyte count, and albumin and higher white blood cell count, neutrophil count, platelet count, NLR, PLR, and SII were associated with recurrence. Sex, alcohol consumption, coronary artery disease, gross hematuria, tumor location, bladder perforation, induction intravesical therapy, and creatinine were not statistically significant in the univariable analyses (**Table 3**).

**Table 3:** Univariable logistic regression analysis of recurrence.

Variable	Unadjusted OR	95% CI	Wald χ²	P value
Age, per 1-year increase	1.04	1.02–1.06	12.10	<0.001
Male sex	1.20	0.82–1.75	0.84	0.359
BMI, per 1 kg/m² increase	1.09	1.03–1.15	8.95	0.003
Smoking history	1.72	1.24–2.40	10.31	0.001
Alcohol consumption history	1.18	0.84–1.66	0.87	0.351
Hypertension	1.46	1.05–2.03	5.11	0.024
Diabetes mellitus	1.51	1.05–2.18	4.88	0.027
Coronary artery disease	1.23	0.80–1.91	0.90	0.342
Previous urinary system disease history	1.64	1.13–2.37	6.93	0.008
Gross hematuria	1.03	0.72–1.47	0.02	0.876
Urinary frequency	1.63	1.16–2.28	8.02	0.004
Urinary urgency	1.57	1.12–2.21	6.91	0.008
Dysuria	1.47	1.02–2.11	4.22	0.039
Other lower urinary tract symptoms	1.14	0.78–1.67	0.44	0.507
Tumor number (multiple vs solitary)	2.12	1.52–2.96	19.51	<0.001
Maximum tumor diameter, per 1-cm increase	1.73	1.47–2.03	40.56	<0.001
Tumor location (multiple vs single location)	0.84	0.56–1.26	0.71	0.398
Pathological stage (T1/primary CIS vs Ta)	1.77	1.27–2.46	11.40	<0.001
High-grade pathology	2.23	1.59–3.13	21.52	<0.001
Concomitant CIS	2.12	1.36–3.31	11.14	<0.001
Multifocality	1.91	1.36–2.69	13.64	<0.001
Operative time, per 1-min increase	1.03	1.01–1.05	11.04	<0.001
Complete resection	0.54	0.30–0.97	4.21	0.038
Obturator nerve reflex	1.93	1.18–3.17	6.91	0.008
Bladder perforation	1.74	0.73–4.17	1.56	0.207
Immediate postoperative intravesical instillation	0.61	0.42–0.88	6.93	0.008
Induction intravesical therapy	0.77	0.53–1.11	2.02	0.154
Maintenance intravesical therapy	0.52	0.37–0.72	14.90	<0.001
Repeat TURBT	1.66	1.07–2.56	5.23	0.021
Hemoglobin, per 1 g/L increase	0.98	0.97–0.99	9.46	0.002
White blood cell count, per 1 × 10^9^/L increase	1.13	1.04–1.22	8.52	0.004
Neutrophil count, per 1 × 10^9^/L increase	1.24	1.12–1.38	16.77	<0.001
Lymphocyte count, per 1 × 10^9^/L increase	0.46	0.31–0.69	14.98	<0.001
Platelet count, per 10 × 10^9^/L increase	1.03	1.01–1.06	5.80	0.016
Albumin, per 1 g/L increase	0.92	0.88–0.96	13.32	<0.001
Creatinine, per 1 μmol/L increase	1.01	1.00–1.02	2.90	0.089
NLR, per 1-unit increase	1.38	1.24–1.54	31.82	<0.001
PLR, per 10-unit increase	1.07	1.03–1.11	11.36	<0.001
SII, per 100-unit increase	1.11	1.06–1.16	19.24	<0.001

BMI, body mass index; CI, confidence interval; CIS, carcinoma *in situ*; NLR, neutrophil-to-lymphocyte ratio; OR, odds ratio; PLR, platelet-to-lymphocyte ratio; SII, systemic immune-inflammation index; TURBT, transurethral resection of bladder tumor.

### 3.5 Multivariable logistic regression analysis and collinearity assessment

In the multivariable logistic regression analysis, multiple tumors were associated with higher odds of documented recurrence (adjusted OR, 1.88; 95% CI, 1.27–2.78; P = 0.002). Higher recurrence odds were also associated with each 1-cm increase in maximum tumor diameter (adjusted OR, 1.42; 95% CI, 1.18–1.71; P < 0.001), T1 or primary CIS versus Ta disease (adjusted OR, 1.59; 95% CI, 1.07–2.36; P = 0.022), high-grade pathology (adjusted OR, 1.77; 95% CI, 1.20–2.61; P = 0.004), concomitant CIS (adjusted OR, 1.82; 95% CI, 1.12–2.95; P = 0.015), and each 1-unit increase in NLR (adjusted OR, 1.25; 95% CI, 1.11–1.41; P < 0.001). Maintenance intravesical therapy was associated with lower odds of documented recurrence (adjusted OR, 0.49; 95% CI, 0.34–0.72; P < 0.001). The other covariates included in the model were not statistically significant (**Table 4**).

**Table 4:** Multivariable logistic regression analysis.

Variable	Adjusted OR	95% CI	Wald χ²	P value
Age, per 1-year increase	1.01	0.99–1.03	0.97	0.325
BMI, per 1 kg/m² increase	1.03	0.97–1.10	0.85	0.357
Smoking history	1.23	0.84–1.81	1.12	0.290
Hypertension	1.12	0.76–1.65	0.33	0.567
Diabetes mellitus	1.16	0.76–1.77	0.47	0.491
Previous urinary system disease history	1.25	0.83–1.89	1.13	0.288
Multiple tumors	1.88	1.27–2.78	9.98	0.002
Maximum tumor diameter, per 1-cm increase	1.42	1.18–1.71	13.73	<0.001
Pathological stage (T1/primary CIS vs Ta)	1.59	1.07–2.36	5.28	0.022
High-grade pathology	1.77	1.20–2.61	8.30	0.004
Concomitant CIS	1.82	1.12–2.95	5.88	0.015
Complete resection	0.73	0.38–1.39	0.90	0.341
Immediate postoperative intravesical instillation	0.82	0.55–1.23	0.93	0.334
Maintenance intravesical therapy	0.49	0.34–0.72	13.89	<0.001
Repeat TURBT	1.18	0.73–1.91	0.46	0.500
NLR, per 1-unit increase	1.25	1.11–1.41	13.37	<0.001

BMI, body mass index; CI, confidence interval; CIS, carcinoma *in situ*; NLR, neutrophil-to-lymphocyte ratio; OR, odds ratio; TURBT, transurethral resection of bladder tumor.

Correlation analysis identified moderate-to-strong correlations between WBC and neutrophil count, between NLR and neutrophil count, and between NLR and SII. Multiple tumors and multifocality were also moderately correlated. To reduce mathematical and conceptual redundancy, NLR was retained as the principal inflammatory marker, whereas its component cell counts and related composite inflammatory indices were not entered simultaneously into the final multivariable models. All variance inflation factors were below 2.2, and all tolerance values exceeded 0.45, indicating no evidence of problematic multicollinearity among the retained variables (**Supplementary Tables 2A–C**).

### 3.6 Recurrence-free survival and complementary Cox regression analysis

The estimated recurrence-free survival rates for the overall cohort were 81.5% (95% CI, 78.2%–84.4%) at 1 year, 63.8% (95% CI, 59.6%–67.7%) at 3 years, and 54.1% (95% CI, 48.9%–59.0%) at 5 years. Lower recurrence-free survival was observed among patients with multiple tumors, tumors measuring at least 3 cm, T1 or primary CIS, high-grade pathology, concomitant CIS, and no maintenance intravesical therapy (all log-rank P ≤ 0.001) (**Table 5**). The corresponding Kaplan–Meier curves, censoring indicators, and numbers at risk are shown in **Figure 1**.

**Table 5:** Kaplan–Meier estimates of recurrence-free survival.

Variable	n	Recurrence events, n (%)	No. at risk at 1 year	1-year RFS, % (95% CI)	No. at risk at 3 years	3-year RFS, % (95% CI)	No. at risk at 5 years	5-year RFS, % (95% CI)	Log-rank χ²	P value
Overall cohort	605	237 (39.2)	480	81.5 (78.2–84.4)	286	63.8 (59.6–67.7)	118	54.1 (48.9–59.0)	—	—
Tumor number										
Solitary	303	92 (30.4)	263	88.2 (84.0–91.4)	161	72.6 (67.0–77.4)	72	63.5 (56.5–69.7)	19.84	<0.001
Multiple	302	145 (48.0)	217	74.8 (69.4–79.4)	125	54.8 (48.6–60.6)	46	43.9 (36.7–50.8)	19.84	<0.001
Maximum tumor diameter										
<3 cm	346	106 (30.6)	291	87.1 (83.2–90.2)	182	71.9 (66.7–76.4)	82	62.7 (56.2–68.5)	24.67	<0.001
≥3 cm	259	131 (50.6)	189	73.9 (68.0–78.9)	104	53.1 (46.5–59.3)	36	41.6 (33.8–49.2)	24.67	<0.001
Pathological stage										
Ta	297	96 (32.3)	251	86.5 (82.0–90.0)	157	70.3 (64.5–75.4)	70	60.8 (53.8–67.1)	11.92	0.001
T1/primary CIS	308	141 (45.8)	229	76.8 (71.6–81.2)	129	57.5 (51.4–63.1)	48	47.2 (40.0–54.1)	11.92	0.001
Pathological grade										
Low-grade	273	79 (28.9)	238	88.7 (84.3–92.0)	150	74.1 (68.3–79.0)	68	65.3 (58.2–71.5)	23.44	<0.001
High-grade	332	158 (47.6)	242	75.7 (70.7–80.0)	136	55.8 (50.0–61.3)	50	44.6 (37.9–51.1)	23.44	<0.001
Concomitant CIS										
No	510	185 (36.3)	417	83.9 (80.3–87.0)	250	66.9 (62.4–71.0)	106	57.2 (51.7–62.3)	12.37	<0.001
Yes	95	52 (54.7)	63	68.4 (58.0–76.8)	36	47.6 (36.5–58.0)	12	35.9 (24.5–47.5)	12.37	<0.001
Maintenance intravesical therapy										
No	238	116 (48.7)	171	73.6 (67.3–78.9)	94	53.1 (46.2–59.6)	34	42.0 (34.1–49.7)	16.58	<0.001
Yes	367	121 (33.0)	309	86.6 (82.6–89.8)	192	70.4 (65.3–74.9)	84	61.8 (55.6–67.4)	16.58	<0.001

CI, confidence interval; CIS, carcinoma *in situ*; RFS, recurrence-free survival.

**Figure 1:**
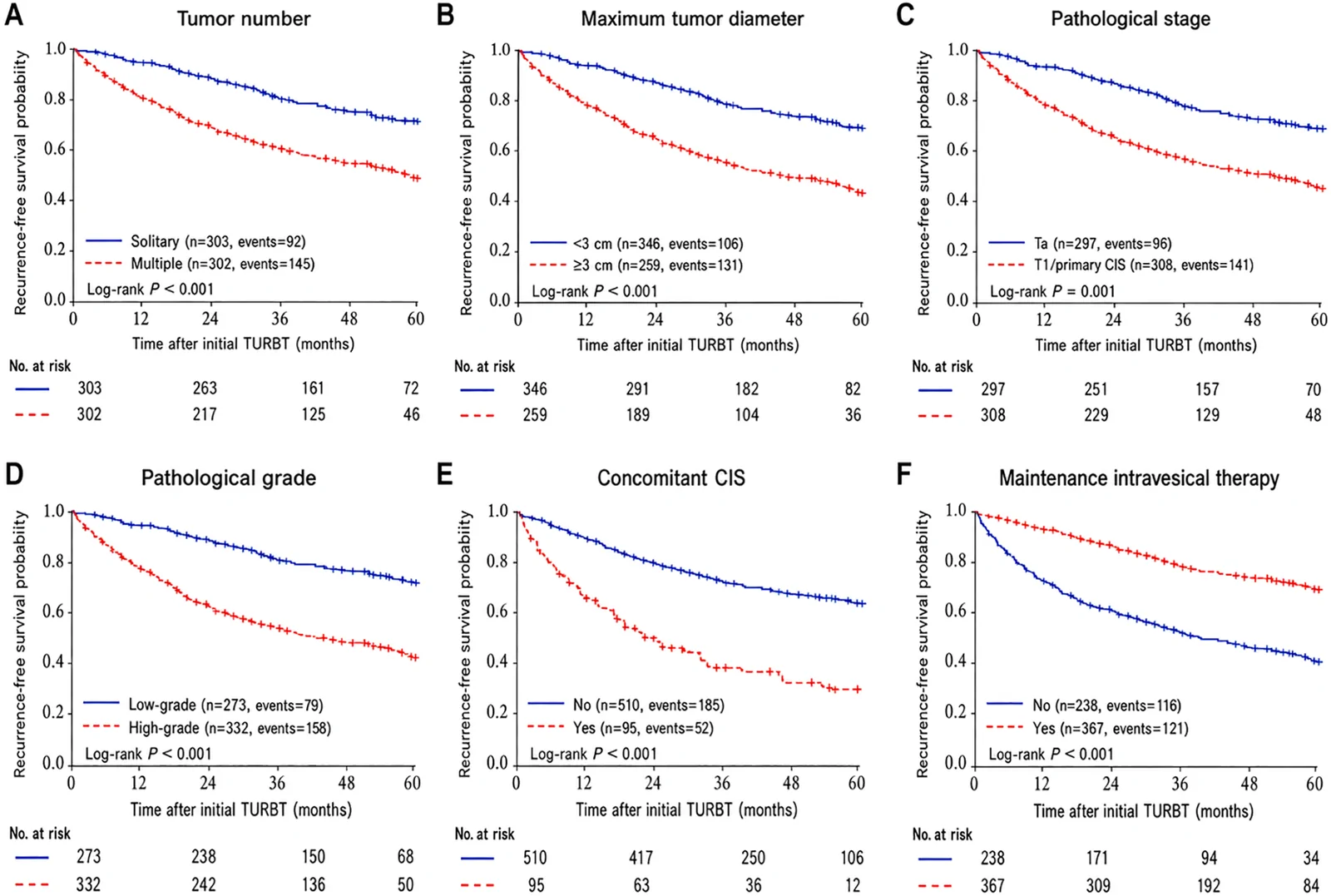
Kaplan–Meier curves of recurrence-free survival according to clinicopathological and treatment-related factors. Recurrence-free survival was compared according to **(A)** tumor number (solitary vs multiple), **(B)** maximum tumor diameter (<3 vs ≥3 cm), **(C)** pathological stage (Ta vs T1/primary CIS), **(D)** pathological grade (low vs high grade), **(E)** concomitant CIS (no vs yes), and **(F)** maintenance intravesical therapy (no vs yes). Tick marks on the curves indicate censored observations. Numbers at risk are presented at baseline and at 1, 3, and 5 years. Between-group differences were assessed using the log-rank test. CIS, carcinoma *in situ*; TURBT, transurethral resection of bladder tumor.

In the complementary multivariable Cox analysis, multiple tumors were associated with a higher recurrence hazard (adjusted HR, 1.62; 95% CI, 1.25–2.10; P < 0.001). Higher hazards were also observed with increasing tumor diameter (adjusted HR per 1-cm increase, 1.24; 95% CI, 1.13–1.36; P < 0.001), T1 or primary CIS (adjusted HR, 1.39; 95% CI, 1.06–1.82; P = 0.017), high-grade pathology (adjusted HR, 1.46; 95% CI, 1.12–1.91; P = 0.005), concomitant CIS (adjusted HR, 1.54; 95% CI, 1.12–2.12; P = 0.008), and increasing NLR (adjusted HR per 1-unit increase, 1.14; 95% CI, 1.07–1.22; P < 0.001). Maintenance intravesical therapy was associated with a lower recurrence hazard (adjusted HR, 0.62; 95% CI, 0.47–0.81; P < 0.001) (**Table 6**).

**Table 6:** Complementary multivariable Cox proportional hazards analysis.

Variable	Adjusted HR	95% CI	Wald χ²	P value
Age, per 1-year increase	1.01	0.99–1.02	1.71	0.191
BMI, per 1 kg/m² increase	1.02	0.98–1.06	0.98	0.323
Smoking history	1.15	0.87–1.52	0.96	0.326
Hypertension	1.08	0.82–1.42	0.30	0.583
Diabetes mellitus	1.12	0.83–1.51	0.55	0.458
Previous urinary system disease history	1.19	0.88–1.61	1.27	0.259
Multiple tumors	1.62	1.25–2.10	13.29	<0.001
Maximum tumor diameter, per 1-cm increase	1.24	1.13–1.36	20.72	<0.001
Pathological stage (T1/primary CIS vs Ta)	1.39	1.06–1.82	5.70	0.017
High-grade pathology	1.46	1.12–1.91	7.72	0.005
Concomitant CIS	1.54	1.12–2.12	7.04	0.008
Complete resection	0.84	0.54–1.31	0.59	0.441
Immediate postoperative intravesical instillation	0.87	0.65–1.16	0.89	0.346
Maintenance intravesical therapy	0.62	0.47–0.81	11.85	<0.001
Repeat TURBT	1.10	0.77–1.57	0.28	0.600
NLR, per 1-unit increase	1.14	1.07–1.22	15.33	<0.001

BMI, body mass index; CI, confidence interval; CIS, carcinoma *in situ*; HR, hazard ratio; NLR, neutrophil-to-lymphocyte ratio; TURBT, transurethral resection of bladder tumor.

The univariable and multivariable Cox results were directionally consistent with those of the primary multivariable logistic regression analysis. There was no evidence that the proportional hazards assumption was violated for any individual covariate or for the model globally (global test, P = 0.412) (**Supplementary Table 3**).

### 3.7 Characteristics of intravesical therapy

Immediate postoperative intravesical instillation was administered to 449 patients, induction therapy to 448, and maintenance therapy to 367. Among patients receiving induction therapy, 176 received a BCG-based regimen and 272 received an intravesical chemotherapy-based regimen. The planned induction course was completed by 396 patients (88.4%).

Among patients who initiated maintenance therapy, 145 received BCG-based treatment and 222 received chemotherapy-based treatment. The median number of maintenance instillations was lower in the recurrence group than in the non-recurrence group, as was the median total treatment duration. Completion of the planned maintenance regimen was less frequent among patients with recurrence, whereas treatment interruption or discontinuation was more frequent. Documented recurrence or progression accounted for 35 of the 138 maintenance-treatment interruptions or discontinuations. Because treatment selection, completion, and discontinuation were related to clinical characteristics and subsequent disease status, these descriptive comparisons were not interpreted as estimates of comparative treatment efficacy (**Supplementary Table 1**).

### 3.8 Subgroup, sensitivity, early-lesion, and follow-up analyses

In analyses stratified by pathological stage, multiple tumors, larger maximum tumor diameter, high-grade pathology, maintenance intravesical therapy, and NLR remained associated with recurrence in both the Ta and T1 subgroups. Concomitant CIS was associated with recurrence in the T1 subgroup but not in the Ta subgroup. No statistically significant interactions were identified between pathological stage and the examined predictors. The 51 patients with primary CIS were excluded from the Ta-versus-T1 interaction analysis and were described separately; 23 of these patients experienced recurrence (**Supplementary Tables 4A, B**).

The magnitudes and directions of the principal adjusted associations were generally maintained after excluding patients who underwent repeat TURBT, excluding 47 non-recurrent patients with less than 12 months of follow-up, and excluding all 29 patients with lesions detected within 3 months after TURBT. In the analysis excluding repeat TURBT, the association for pathological stage was attenuated and did not reach conventional statistical significance, whereas the remaining principal associations were maintained (**Supplementary Table 5**).

Among the 29 patients with lesions detected within 3 months, 18 lesions occurred at the original tumor site and 11 occurred at a different site. Seventeen patients underwent repeat TURBT, with persistent tumor identified in 9. After review of operative, pathological, and follow-up records, 10 lesions were classified as residual disease, 16 as recurrence, and 3 remained uncertain. Only the 16 lesions adjudicated as recurrence were counted as recurrence events in the primary analysis (**Supplementary Table 6**).

Among the 47 patients without recurrence who had less than 12 months of follow-up, the documented reasons for early termination included non-bladder-cancer death, transfer to another institution, relocation, severe unrelated illness, refusal of further surveillance, and other documented reasons; none were classified as unable to contact. Baseline characteristics were generally similar between patients with shorter and longer follow-up, with absolute standardized mean differences ranging from 0.01 to 0.19 (**Supplementary Tables 7A, B**).

The median time to the first postoperative cystoscopy was 3.1 months, and patients underwent a median of 6 cystoscopies during follow-up. Patients with high-grade disease underwent earlier and more frequent cystoscopic surveillance than those with low-grade disease. Delayed or missed surveillance visits were recorded in 128 patients (21.2%) (**Supplementary Table 8**).

### 3.9 Association pattern and time-dependent discrimination of baseline NLR

Restricted cubic spline analysis based on the complementary Cox model showed that baseline NLR was associated with recurrence hazard (P for overall association < 0.001), with no statistically significant evidence of nonlinearity (P for nonlinearity = 0.214). Relative to the reference value of NLR = 2.0, higher NLR values were associated with progressively higher adjusted hazards of recurrence (**Supplementary Figure 1**).

The time-dependent discriminatory performance of baseline NLR decreased modestly over time. The time-dependent AUC was 0.660 (95% CI, 0.619–0.701) at 1 year, 0.642 (95% CI, 0.598–0.686) at 3 years, and 0.623 (95% CI, 0.568–0.678) at 5 years. The corresponding data-derived exploratory cutoffs were 2.89, 2.78, and 2.70, respectively. These cutoffs were considered exploratory and were not interpreted as validated clinical thresholds (**Supplementary Table 9**). The corresponding time-dependent ROC curves are presented in **Supplementary Figure 2**.

### 3.10 Discrimination and calibration of the multivariable models

The multivariable logistic model yielded an apparent AUC of 0.785 and a bootstrap-corrected AUC of 0.769 (95% CI, 0.748–0.820). The bootstrap-corrected calibration intercept, calibration slope, and Brier score were −0.021, 0.921, and 0.183, respectively. The complementary Cox model yielded an apparent Harrell’s C-index of 0.755 and a bootstrap-corrected C-index of 0.739 (95% CI, 0.710–0.771). Its bootstrap-corrected time-dependent AUCs were 0.758, 0.734, and 0.721 at 1, 3, and 5 years, respectively (**Supplementary Tables 10A, B**). The apparent and bootstrap-corrected calibration curves for the multivariable logistic model are presented in **Supplementary Figure 3**.

### 3.11 Exploratory analyses of TURBT quality parameters

Information on detrusor muscle status, resection technique, and imaging modality at initial TURBT was available for 558 (92.2%), 517 (85.5%), and 492 (81.3%) patients, respectively. Among patients with available pathology reports, detrusor muscle was present in 449 (80.5%), absent in 87 (15.6%), and not assessable in 22 (3.9%), with no significant difference in its distribution according to recurrence status (P = 0.150). En bloc resection was performed in 112 of 517 patients (21.7%), and enhanced imaging was used in 91 of 492 patients (18.5%); neither parameter differed significantly between the recurrence and non-recurrence groups (P = 0.180 and P = 0.168, respectively). Repeat TURBT was performed in 99 patients (16.4%) at a median of 42 days after initial TURBT. Residual tumor was identified in 40 patients (40.4%) and was more frequent in the recurrence group than in the non-recurrence group (51.0% vs 30.0%; P = 0.033). Upstaging occurred in 8 patients (8.1%), without a statistically significant between-group difference (P = 0.160) (**Supplementary Table 11**).

## 4 Discussion

In this retrospective cohort of 605 patients with pathologically confirmed NMIBC after initial TURBT, 39.2% developed documented intravesical recurrence during a median follow-up of 34 months. Multiple tumors, increasing tumor diameter, T1 or primary CIS, high-grade pathology, concomitant CIS, and higher baseline NLR were independently associated with recurrence in both the primary logistic model and the complementary Cox model, whereas maintenance intravesical therapy was associated with lower recurrence odds and hazard. The estimated recurrence-free survival rates declined from 81.5% at 1 year to 63.8% at 3 years and 54.1% at 5 years, underscoring the sustained recurrence burden after initial resection. A notable contribution of this study is the integration of routinely available clinicopathological, treatment-related, inflammatory, follow-up, and TURBT-quality information within a consistent analytical framework that included early-lesion adjudication, sensitivity analyses, time-to-event modeling, collinearity assessment, and bootstrap-corrected model evaluation. Collectively, these findings support a multidimensional approach to postoperative risk assessment and may help identify patients who warrant closer surveillance and more rigorous treatment monitoring, while remaining associative rather than causal (19).

The clinicopathological pattern observed in this cohort suggests that postoperative recurrence risk is characterized by several related but non-equivalent dimensions of NMIBC. Tumor number and maximum diameter primarily reflect the extent of visible intravesical disease, whereas pathological stage, grade, and CIS provide information regarding tissue invasion and histological aggressiveness (20, 21). Their simultaneous retention in the adjusted models indicates that tumor burden and pathological phenotype each contributed distinct prognostic information. The clear separation of the Kaplan–Meier curves further illustrates the cumulative clinical relevance of these features over time, with particularly unfavorable recurrence-free survival observed among patients with multiple tumors, larger lesions, high-grade disease, or concomitant CIS. These findings are compatible with the concept that patients with a broader urothelial tumor field or more biologically aggressive pathological characteristics may remain at elevated risk beyond the immediate postoperative period (22, 23). The stage-stratified analyses provide additional context for interpreting these associations. Tumor number, diameter, grade, NLR, and maintenance intravesical therapy showed broadly similar associations in the Ta and T1 subgroups, and no statistically significant interactions with pathological stage were identified. This pattern suggests that the prognostic information conveyed by these variables was not restricted to a single stage category within the present cohort. Concomitant CIS was associated with recurrence in the T1 subgroup but not in the Ta subgroup; however, the nonsignificant interaction test does not support a definitive stage-specific difference. In contrast, several demographic characteristics, comorbidities, urinary symptoms, and perioperative variables were associated with recurrence in univariable analyses but were attenuated after multivariable adjustment. This attenuation suggests that some crude associations may have reflected differences in tumor characteristics, treatment patterns, or correlated clinical factors rather than independent prognostic contributions. Taken together, these results support the continued use of tumor number, size, stage, grade, and CIS as complementary components of postoperative risk assessment rather than reliance on any single clinicopathological feature (24).

Baseline NLR was associated with recurrence independently of conventional clinicopathological factors. Restricted cubic spline analysis indicated an approximately linear association, supporting its treatment as a continuous variable. However, the time-dependent areas under the curve for NLR alone were modest and declined over time, indicating that NLR should be regarded as an adjunctive marker rather than a standalone prognostic tool or a basis for a definitive clinical cutoff. Although elevated NLR may reflect an imbalance between neutrophil-predominant inflammation and lymphocyte-mediated immune activity, the present findings do not establish a direct biological mechanism (25). Maintenance intravesical therapy was consistently associated with a more favorable recurrence profile across the principal and sensitivity analyses. Patients without recurrence also more frequently completed maintenance treatment and received a greater number and longer duration of instillations. Because treatment allocation and continuation were determined in routine practice and could be influenced by tolerance and disease status, these associations should not be interpreted as causal estimates of treatment efficacy. Surgical technique and resection quality were also explored. Paciotti et al. (26)reported favorable long-term outcomes after en bloc resection, with detrusor muscle identified in all specimens and a 5-year recurrence-free survival rate of 77%. In our available-case analysis, recurrence status did not differ significantly according to en bloc versus conventional TURBT, detrusor muscle status, or enhanced imaging use. These differences may reflect variation in patient selection, tumor-risk composition, treatment, and study design. Residual tumor was identified in 40.4% of patients undergoing repeat TURBT and was more frequent in the recurrence group. Early postoperative lesions comprised residual disease, recurrence, and uncertain cases, underscoring the importance of integrating operative, pathological, and follow-up information when classifying early events.

The present findings are consistent with recent evidence indicating that postoperative recurrence in NMIBC is associated with tumor burden, pathological characteristics, treatment exposure, and routine laboratory markers. Li et al. (27) identified pathological and treatment-related factors associated with 2-year recurrence after TURBT, although their lower recurrence rate may reflect differences in follow-up, patient risk, treatment patterns, and outcome ascertainment. Tang et al. (13) likewise reported associations involving tumor number and routine blood and urinary parameters. In our cohort, multiple tumors, larger tumor diameter, T1 or primary CIS, high-grade pathology, and concomitant CIS remained associated with recurrence after adjustment. The association between baseline NLR and recurrence was also consistent with the meta-analysis by Huang et al. (28) Whereas previous studies mainly used study-specific NLR cutoffs, our spline analysis supported retaining NLR as a continuous variable. Scilipoti et al. (29) reported more favorable RFS with adequate BCG exposure, consistent with the inverse observational association between maintenance intravesical therapy and recurrence in our cohort. The residual-tumor rate after repeat TURBT was also comparable to that reported by Guigui et al. (30) Treatment- and procedure-related findings should nevertheless be interpreted cautiously because of treatment selection, variable treatment completion, and the observational nature of the available evidence. These findings may also be interpreted in the context of established risk stratification systems. The European Organization for Research and Treatment of Cancer (EORTC) and Club Urológico Español de Tratamiento Oncológico (CUETO) models similarly emphasize key clinicopathological factors, including tumor number, tumor size, pathological stage, grade, and CIS. The consistency of our findings with these established domains reinforces their clinical relevance in postoperative recurrence assessment. Although formal model comparison was beyond the scope of this study, NLR may provide complementary information alongside conventional clinicopathological characteristics and warrants further evaluation as an adjunctive marker. The present findings are therefore intended to complement, rather than replace, established risk stratification systems.

The principal associations were directionally consistent between the primary logistic regression and complementary Cox proportional hazards analyses. The logistic model evaluated whether recurrence was documented during the observed follow-up, consistent with the primary study objective, whereas the Cox model incorporated recurrence timing and censoring and provided complementary information on recurrence hazard over time. The two models therefore addressed related but distinct estimands, and their odds ratios and HRs were not interpreted as interchangeable. Cox regression was retained as a complementary analysis because the primary analytical objective focused on overall documented recurrence status rather than recurrence timing. In addition, postoperative treatment exposure, particularly maintenance intravesical therapy, varied in initiation, duration, completion, and discontinuation. Because neither model fully captured these time-varying treatment patterns or fully addressed treatment-selection bias and reverse causation, treatment-related estimates from both analyses require cautious interpretation.

Several limitations should be acknowledged. First, the retrospective single-center design is susceptible to residual confounding and uncertainty related to heterogeneous follow-up. Forty-seven patients without documented recurrence had less than 12 months of follow-up; however, their baseline characteristics were broadly comparable with those of patients followed longer, and their exclusion did not materially alter the principal estimates. Nevertheless, the 5-year estimates should be interpreted cautiously because the number of patients remaining at risk decreased over time. Second, postoperative treatment allocation was non-randomized, and detailed information on dose modifications and the timing and indications for repeat TURBT could not be reconstructed uniformly. Treatment-related associations should therefore not be interpreted causally. Third, data on detrusor muscle, resection technique, and enhanced imaging were incomplete and were analyzed on an exploratory available-case basis. Procedural standardization of all cystoscopic examinations could not be verified, leaving the possibility of information and detection bias. External validation was not performed despite bootstrap correction of model performance. Finally, progression-related outcomes were not systematically assessed; therefore, the findings should be interpreted only in relation to recurrence and should not be extrapolated to progression prediction. Future prospective multicenter studies should standardize surveillance and treatment-exposure assessment, collect complete procedural data, externally validate these findings, and evaluate recurrence and progression concurrently over longer follow-up.

## 5 Conclusion

In conclusion, recurrence after initial TURBT in patients with NMIBC was independently associated with multiple tumors, increasing tumor diameter, T1 or primary CIS, high-grade pathology, concomitant CIS, and higher baseline NLR. Maintenance intravesical therapy was inversely associated with the odds and hazard of recurrence. These findings underscore the complementary relevance of tumor burden, pathological characteristics, systemic inflammatory status, and postoperative treatment exposure in recurrence assessment. These routinely available factors may support postoperative risk stratification and risk-adapted surveillance. However, the treatment-related associations should be interpreted cautiously and require confirmation in prospective studies.

## Data Availability

The raw data supporting the conclusions of this article will be made available by the authors, without undue reservation.
