# Supplementary material for: Risk factors for recurrence after initial transurethral resection of bladder tumor in patients with non–muscle-invasive bladder cancer
Source: Front Oncol. 2026 Oct 1;16:1861461. doi: 10.3389/fonc.2026.1861461 (PMC13668579; doi:10.3389/fonc.2026.1861461)
Supplement: Supplementary file 1 [file SupplementaryFile1.docx]

Supplementary Table S1. Detailed characteristics of intravesical therapy

| Treatment characteristic | Overall | Recurrence group | Non-recurrence group | Available data, n/N (%) | P value |
| --- | --- | --- | --- | --- | --- |
| Immediate postoperative instillation |  |  |  |  |  |
| Received, n (%) | 449 (74.2) | 162 (68.4) | 287 (78.0) | 605/605 (100.0) | 0.008 |
| Agent class |  |  |  | 449/449 (100.0) | 0.357 |
| Pirarubicin, n (%) | 232 (51.7) | 91 (56.2) | 141 (49.1) |  |  |
| Epirubicin, n (%) | 105 (23.4) | 37 (22.8) | 68 (23.7) |  |  |
| Mitomycin C, n (%) | 94 (20.9) | 30 (18.5) | 64 (22.3) |  |  |
| Other documented agent, n (%) | 18 (4.0) | 4 (2.5) | 14 (4.9) |  |  |
| Induction intravesical therapy |  |  |  |  |  |
| Received induction, n (%) | 448 (74.0) | 168 (70.9) | 280 (76.1) | 605/605 (100.0) | 0.154 |
| Regimen class |  |  |  | 448/448 (100.0) | 0.022 |
| BCG-based, n (%) | 176 (39.3) | 78 (46.4) | 98 (35.0) |  |  |
| Chemotherapy-based, n (%) | 272 (60.7) | 90 (53.6) | 182 (65.0) |  |  |
| Actual number of instillations, median (IQR) | 6 (6–8) | 6 (5–8) | 6 (6–8) | 421/448 (94.0) | 0.183 |
| Completed planned induction, n (%) | 396 (88.4) | 142 (84.5) | 254 (90.7) | 448/448 (100.0) | 0.048 |
| Maintenance intravesical therapy |  |  |  |  |  |
| Initiated maintenance, n (%) | 367 (60.7) | 121 (51.1) | 246 (66.8) | 605/605 (100.0) | <0.001 |
| Regimen class |  |  |  | 367/367 (100.0) | 0.028 |
| BCG-based, n (%) | 145 (39.5) | 58 (47.9) | 87 (35.4) |  |  |
| Chemotherapy-based, n (%) | 222 (60.5) | 63 (52.1) | 159 (64.6) |  |  |
| Actual number of maintenance instillations, median (IQR) | 10 (6–15) | 8 (5–12) | 11 (7–16) | 338/367 (92.1) | <0.001 |
| Total treatment duration, months, median (IQR) | 9 (6–14) | 7 (4–11) | 10 (7–15) | 331/367 (90.2) | <0.001 |
| Completed planned regimen, n (%) | 229 (62.4) | 58 (47.9) | 171 (69.5) | 367/367 (100.0) | <0.001 |
| Interruption or discontinuation, n (%) | 138 (37.6) | 63 (52.1) | 75 (30.5) | 367/367 (100.0) | <0.001 |
| Reason for interruption or discontinuation |  |  |  | 138/138 (100.0) | <0.001 |
| Treatment-related toxicity, n (%) | 45 (32.6) | 18 (28.6) | 27 (36.0) |  |  |
| Documented recurrence or progression, n (%) | 35 (25.4) | 35 (55.6) | 0 (0.0) |  |  |
| Suspected recurrence or progression not subsequently confirmed, n (%) | 6 (4.3) | 0 (0.0) | 6 (8.0) |  |  |
| Patient preference, n (%) | 25 (18.1) | 6 (9.5) | 19 (25.3) |  |  |
| Drug availability or logistical reason, n (%) | 15 (10.9) | 2 (3.2) | 13 (17.3) |  |  |
| Other documented reason, n (%) | 12 (8.7) | 2 (3.2) | 10 (13.3) |  |  |

Categorical variables were compared using the Pearson chi-square test, continuity-corrected chi-square test, or Fisher’s exact test, as appropriate.

Abbreviations: BCG, Bacillus Calmette–Guérin; IQR, interquartile range.

Supplementary Table S2A. Spearman correlation matrix for inflammatory markers

| Variable | WBC | Neutrophils | Lymphocytes | Platelets | NLR | PLR | SII |
| --- | --- | --- | --- | --- | --- | --- | --- |
| WBC | 1.00 | 0.77 | −0.08 | 0.19 | 0.57 | 0.13 | 0.60 |
| Neutrophils | 0.77 | 1.00 | −0.18 | 0.18 | 0.70 | 0.19 | 0.69 |
| Lymphocytes | −0.08 | −0.18 | 1.00 | 0.04 | −0.60 | −0.53 | −0.39 |
| Platelets | 0.19 | 0.18 | 0.04 | 1.00 | 0.10 | 0.53 | 0.55 |
| NLR | 0.57 | 0.70 | −0.60 | 0.10 | 1.00 | 0.48 | 0.72 |
| PLR | 0.13 | 0.19 | −0.53 | 0.53 | 0.48 | 1.00 | 0.64 |
| SII | 0.60 | 0.69 | −0.39 | 0.55 | 0.72 | 0.64 | 1.00 |

Abbreviations: NLR, neutrophil-to-lymphocyte ratio; PLR, platelet-to-lymphocyte ratio; SII, systemic immune-inflammation index; WBC, white blood cell count.

Supplementary Table S2B. Correlation among clinicopathological variables

| Variable | Multiple tumors | Multifocality | T1/primary CIS | High grade | Concomitant CIS |
| --- | --- | --- | --- | --- | --- |
| Multiple tumors | 1.00 | 0.68 | 0.24 | 0.19 | 0.15 |
| Multifocality | 0.68 | 1.00 | 0.21 | 0.17 | 0.18 |
| T1/primary CIS | 0.24 | 0.21 | 1.00 | 0.51 | 0.37 |
| High grade | 0.19 | 0.17 | 0.51 | 1.00 | 0.32 |
| Concomitant CIS | 0.15 | 0.18 | 0.37 | 0.32 | 1.00 |

Abbreviation: CIS, carcinoma in situ.

Supplementary Table S2C. VIF and tolerance statistics

| Variable | Tolerance | VIF |
| --- | --- | --- |
| Age | 0.91 | 1.10 |
| BMI | 0.89 | 1.12 |
| Smoking history | 0.87 | 1.15 |
| Hypertension | 0.84 | 1.19 |
| Diabetes mellitus | 0.90 | 1.11 |
| Previous urinary system disease history | 0.93 | 1.08 |
| Multiple tumors | 0.53 | 1.89 |
| Maximum tumor diameter | 0.72 | 1.39 |
| Pathological stage | 0.49 | 2.04 |
| High-grade pathology | 0.46 | 2.17 |
| Concomitant CIS | 0.66 | 1.52 |
| Complete resection | 0.88 | 1.14 |
| Immediate postoperative intravesical instillation | 0.82 | 1.22 |
| Maintenance intravesical therapy | 0.74 | 1.35 |
| Repeat TURBT | 0.79 | 1.27 |
| NLR | 0.86 | 1.16 |

Abbreviations: BMI, body mass index; CIS, carcinoma in situ; NLR, neutrophil-to-lymphocyte ratio; TURBT, transurethral resection of bladder tumor; VIF, variance inflation factor.

Supplementary Table S3. Full Cox proportional hazards regression analysis

| Variable | Univariable HR | 95% CI | P value | Adjusted HR | 95% CI | P value | PH-assumption P value |
| --- | --- | --- | --- | --- | --- | --- | --- |
| Age, per 1-year increase | 1.03 | 1.01–1.05 | 0.003 | 1.01 | 0.99–1.02 | 0.191 | 0.120 |
| Male sex | 1.13 | 0.82–1.56 | 0.456 | — | — | — | 0.193 |
| BMI, per 1 kg/m² increase | 1.06 | 1.01–1.11 | 0.016 | 1.02 | 0.98–1.06 | 0.323 | 0.266 |
| Smoking history | 1.42 | 1.09–1.85 | 0.009 | 1.15 | 0.87–1.52 | 0.326 | 0.339 |
| Alcohol consumption history | 1.11 | 0.84–1.47 | 0.465 | — | — | — | 0.412 |
| Hypertension | 1.27 | 0.98–1.64 | 0.069 | 1.08 | 0.82–1.42 | 0.583 | 0.485 |
| Diabetes mellitus | 1.31 | 0.98–1.74 | 0.065 | 1.12 | 0.83–1.51 | 0.458 | 0.558 |
| Coronary artery disease | 1.14 | 0.82–1.58 | 0.434 | — | — | — | 0.631 |
| Previous urinary system disease history | 1.38 | 1.04–1.84 | 0.027 | 1.19 | 0.88–1.61 | 0.259 | 0.704 |
| Gross hematuria | 1.02 | 0.78–1.33 | 0.884 | — | — | — | 0.777 |
| Urinary frequency | 1.34 | 1.03–1.74 | 0.029 | — | — | — | 0.850 |
| Urinary urgency | 1.31 | 1.01–1.71 | 0.044 | — | — | — | 0.143 |
| Dysuria | 1.25 | 0.94–1.66 | 0.124 | — | — | — | 0.216 |
| Other lower urinary tract symptoms | 1.09 | 0.81–1.46 | 0.566 | — | — | — | 0.289 |
| Tumor number, multiple vs solitary | 1.69 | 1.30–2.19 | <0.001 | 1.62 | 1.25–2.10 | <0.001 | 0.362 |
| Maximum tumor diameter, per 1-cm increase | 1.34 | 1.23–1.46 | <0.001 | 1.24 | 1.13–1.36 | <0.001 | 0.435 |
| Tumor location, multiple vs single location | 0.91 | 0.67–1.25 | 0.553 | — | — | — | 0.508 |
| Pathological stage, T1/primary CIS vs Ta | 1.53 | 1.18–1.98 | 0.001 | 1.39 | 1.06–1.82 | 0.017 | 0.581 |
| High-grade pathology | 1.67 | 1.28–2.18 | <0.001 | 1.46 | 1.12–1.91 | 0.005 | 0.654 |
| Concomitant CIS | 1.73 | 1.27–2.36 | <0.001 | 1.54 | 1.12–2.12 | 0.008 | 0.727 |
| Multifocality | 1.54 | 1.18–2.01 | 0.001 | — | — | — | 0.800 |
| Operative time, per 1-min increase | 1.02 | 1.01–1.03 | <0.001 | — | — | — | 0.873 |
| Complete resection | 0.7 | 0.48–1.03 | 0.067 | 0.84 | 0.54–1.31 | 0.441 | 0.166 |
| Obturator nerve reflex | 1.52 | 1.08–2.14 | 0.016 | — | — | — | 0.239 |
| Bladder perforation | 1.35 | 0.74–2.46 | 0.327 | — | — | — | 0.312 |
| Immediate postoperative intravesical instillation | 0.75 | 0.57–0.98 | 0.037 | 0.87 | 0.65–1.16 | 0.346 | 0.385 |
| Induction intravesical therapy | 0.84 | 0.64–1.10 | 0.207 | — | — | — | 0.458 |
| Maintenance intravesical therapy | 0.63 | 0.49–0.81 | <0.001 | 0.62 | 0.47–0.81 | <0.001 | 0.531 |
| Repeat TURBT | 1.32 | 0.98–1.77 | 0.066 | 1.1 | 0.77–1.57 | 0.600 | 0.604 |
| Hemoglobin, per 1 g/L increase | 0.99 | 0.98–1.00 | 0.051 | — | — | — | 0.677 |
| White blood cell count, per 1 × 10⁹/L increase | 1.08 | 1.02–1.14 | 0.007 | — | — | — | 0.750 |
| Neutrophil count, per 1 × 10⁹/L increase | 1.16 | 1.08–1.24 | <0.001 | — | — | — | 0.823 |
| Lymphocyte count, per 1 × 10⁹/L increase | 0.62 | 0.44–0.87 | 0.006 | — | — | — | 0.896 |
| Platelet count, per 10 × 10⁹/L increase | 1.02 | 0.99–1.05 | 0.187 | — | — | — | 0.189 |
| Albumin, per 1 g/L increase | 0.95 | 0.92–0.98 | 0.001 | — | — | — | 0.262 |
| Creatinine, per 1 μmol/L increase | 1 | 0.99–1.01 | 1.000 | — | — | — | 0.335 |
| NLR, per 1-unit increase | 1.19 | 1.12–1.27 | <0.001 | 1.14 | 1.07–1.22 | <0.001 | 0.408 |
| PLR, per 10-unit increase | 1.04 | 1.01–1.07 | 0.008 | — | — | — | 0.481 |
| SII, per 100-unit increase | 1.07 | 1.04–1.11 | <0.001 | — | — | — | 0.554 |
| Global proportional hazards test | — | — | — | — | — | — | 0.412 |

Abbreviations: BMI, body mass index; CI, confidence interval; CIS, carcinoma in situ; HR, hazard ratio; NLR, neutrophil-to-lymphocyte ratio; PH, proportional hazards; PLR, platelet-to-lymphocyte ratio; SII, systemic immune-inflammation index; TURBT, transurethral resection of bladder tumor.

Supplementary Table S4A. Pathological-stage subgroup analysis

| Variable | Ta subgroup, adjusted OR (95% CI) | P value | T1 subgroup, adjusted OR (95% CI) | P value | P for interaction |
| --- | --- | --- | --- | --- | --- |
| Multiple tumors | 1.74 (1.05–2.88) | 0.031 | 1.91 (1.14–3.21) | 0.014 | 0.732 |
| Maximum tumor diameter, per 1-cm increase | 1.36 (1.08–1.71) | 0.008 | 1.47 (1.14–1.88) | 0.003 | 0.681 |
| High-grade pathology | 1.68 (1.01–2.79) | 0.046 | 1.83 (1.11–3.02) | 0.018 | 0.824 |
| Concomitant CIS | 1.39 (0.74–2.62) | 0.307 | 2.02 (1.12–3.66) | 0.020 | 0.214 |
| Maintenance intravesical therapy | 0.56 (0.34–0.91) | 0.020 | 0.47 (0.28–0.79) | 0.004 | 0.598 |
| NLR, per 1-unit increase | 1.19 (1.02–1.40) | 0.031 | 1.28 (1.08–1.51) | 0.004 | 0.557 |

Ta n = 297; T1 n = 257. The 51 patients with primary CIS are excluded from the Ta-versus-T1 interaction analysis and described separately.

Abbreviations: CI, confidence interval; CIS, carcinoma in situ; NLR, neutrophil-to-lymphocyte ratio; OR, odds ratio.

Staging definitions: Ta, noninvasive papillary carcinoma; T1, tumor invading the subepithelial connective tissue.

Supplementary Table S4B. Descriptive characteristics of primary CIS

| Characteristic | Primary CIS group (n = 51) |
| --- | --- |
| Recurrence events, n (%) | 23 (45.1) |
| Follow-up duration, months, median (IQR) | 31 (20–47) |
| Time to recurrence among recurrent cases, months, median (IQR) | 13 (7–22) |
| High-grade pathology, n (%) | 51 (100.0) |
| Maintenance intravesical therapy, n (%) | 36 (70.6) |
| NLR | 3.05 ± 1.32 |

Abbreviations: CIS, carcinoma in situ; IQR, interquartile range; NLR, neutrophil-to-lymphocyte ratio.

Supplementary Table S5. Sensitivity analyses of factors associated with recurrence

| Variable | Primary logistic model, n=605 | Excluding repeat TURBT, n=506 | Excluding follow-up <12 months without recurrence, n=558 | Excluding all patients with lesions detected ≤3 months, n=576; 221 events |
| --- | --- | --- | --- | --- |
| Multiple tumors | 1.88 (1.27–2.78); P=0.002 | 1.79 (1.17–2.74); P=0.007 | 1.83 (1.22–2.73); P=0.003 | 1.82 (1.21–2.75); P=0.004 |
| Maximum tumor diameter, per 1-cm increase | 1.42 (1.18–1.71); P<0.001 | 1.38 (1.12–1.69); P=0.002 | 1.43 (1.18–1.74); P<0.001 | 1.39 (1.15–1.68); P=0.001 |
| Pathological stage, T1/primary CIS vs Ta | 1.59 (1.07–2.36); P=0.022 | 1.49 (0.98–2.28); P=0.063 | 1.61 (1.07–2.41); P=0.022 | 1.52 (1.01–2.30); P=0.046 |
| High-grade pathology | 1.77 (1.20–2.61); P=0.004 | 1.69 (1.11–2.56); P=0.014 | 1.76 (1.18–2.62); P=0.005 | 1.70 (1.14–2.54); P=0.010 |
| Concomitant CIS | 1.82 (1.12–2.95); P=0.015 | 1.73 (1.03–2.92); P=0.039 | 1.82 (1.11–2.98); P=0.018 | 1.75 (1.06–2.90); P=0.029 |
| Maintenance intravesical therapy | 0.49 (0.34–0.72); P<0.001 | 0.52 (0.35–0.79); P=0.002 | 0.48 (0.32–0.71); P<0.001 | 0.51 (0.34–0.76); P=0.001 |
| NLR, per 1-unit increase | 1.25 (1.11–1.41); P<0.001 | 1.21 (1.05–1.39); P=0.007 | 1.26 (1.11–1.43); P<0.001 | 1.22 (1.08–1.39); P=0.002 |

Values are presented as adjusted ORs (95% CIs) with corresponding P values.

Abbreviations: CI, confidence interval; CIS, carcinoma in situ; NLR, neutrophil-to-lymphocyte ratio; OR, odds ratio; TURBT, transurethral resection of bladder tumor.

Supplementary Table S6. Adjudication of lesions detected within 3 months after TURBT

| Early-lesion characteristic | n/N (%) |
| --- | --- |
| Lesions detected ≤3 months after TURBT | 29/29 (100.0) |
| Detected at the first postoperative cystoscopy | 26/29 (89.7) |
| Same site as the initial tumor | 18/29 (62.1) |
| Different site from the initial tumor | 11/29 (37.9) |
| Initial resection documented as complete | 22/29 (75.9) |
| Initial resection incomplete or uncertain | 7/29 (24.1) |
| Repeat TURBT performed | 17/29 (58.6) |
| Persistent tumor identified at repeat TURBT | 9/17 (52.9) |
| Adjudicated as residual disease | 10/29 (34.5) |
| Adjudicated as true recurrence | 16/29 (55.2) |
| Classification uncertain | 3/29 (10.3) |
| Counted as recurrence events in the primary analysis | 16/29 (55.2) |
| Not counted as recurrence events in the primary analysis | 13/29 (44.8) |
| Patients excluded from the ≤3-month sensitivity analysis | 29/29 (100.0) |
| Recurrence events removed in the sensitivity analysis | 16/237 (6.8) |

Abbreviation: TURBT, transurethral resection of bladder tumor.

Supplementary Table S7A. Reasons for follow-up shorter than 12 months among patients without recurrence

| Reason for early follow-up termination | n (%) |
| --- | --- |
| Non-bladder-cancer death | 8 (17.0) |
| Transfer to another institution | 12 (25.5) |
| Relocation | 9 (19.1) |
| Severe unrelated illness | 7 (14.9) |
| Patient refusal of further surveillance | 6 (12.8) |
| Unable to contact | 0 (0.0) |
| Other documented reason | 5 (10.6) |
| Total | 47 (100.0) |

Supplementary Table S7B. Baseline characteristics according to early censoring among patients without recurrence

| Variable | Follow-up <12 months without recurrence, n=47 | Follow-up ≥12 months without recurrence, n=321 | Absolute SMD | P value |
| --- | --- | --- | --- | --- |
| Age, years | 65.4 ± 9.6 | 63.7 ± 8.6 | 0.19 | 0.256 |
| Male sex, n (%) | 35 (74.5) | 237 (73.8) | 0.01 | 0.926 |
| Multiple tumors, n (%) | 21 (44.7) | 136 (42.4) | 0.05 | 0.765 |
| Maximum tumor diameter, cm | 2.5 ± 1.1 | 2.4 ± 1.0 | 0.10 | 0.558 |
| Pathological stage: T1/primary CIS, n (%) | 22 (46.8) | 145 (45.2) | 0.03 | 0.833 |
| High-grade pathology, n (%) | 23 (48.9) | 151 (47.0) | 0.04 | 0.808 |
| Concomitant CIS, n (%) | 6 (12.8) | 37 (11.5) | 0.04 | 0.805 |
| Multifocality, n (%) | 13 (27.7) | 90 (28.0) | 0.01 | 0.957 |
| NLR | 2.70 ± 1.25 | 2.63 ± 1.21 | 0.06 | 0.720 |
| Immediate postoperative intravesical instillation, n (%) | 35 (74.5) | 252 (78.5) | 0.10 | 0.533 |
| Maintenance intravesical therapy, n (%) | 29 (61.7) | 217 (67.6) | 0.12 | 0.422 |

Abbreviations: CIS, carcinoma in situ; NLR, neutrophil-to-lymphocyte ratio; SMD, standardized mean difference.

Supplementary Table S8. Follow-up adherence and surveillance intensity according to pathological grade

| Follow-up characteristic | Overall cohort, n=605 | Low-grade disease, n=273 | High-grade disease, n=332 | P value |
| --- | --- | --- | --- | --- |
| Time to first postoperative cystoscopy, months, median (IQR) | 3.1 (2.8–3.6) | 3.3 (2.9–3.8) | 2.9 (2.6–3.4) | <0.001 |
| Number of cystoscopies per patient, median (IQR) | 6 (4–9) | 5 (4–8) | 7 (5–10) | <0.001 |
| Cystoscopies per patient-year, median (IQR) | 2.1 (1.6–2.8) | 1.8 (1.4–2.3) | 2.4 (1.8–3.0) | <0.001 |
| Delayed or missed surveillance visit, n (%) | 128 (21.2) | 48 (17.6) | 80 (24.1) | 0.052 |

Abbreviation: IQR, interquartile range.

Supplementary Table S9. Time-dependent ROC analysis of baseline NLR for recurrence-free survival

| Time point | No. at risk | Time-dependent AUC | 95% CI | Exploratory cutoff | Sensitivity, % | Specificity, % |
| --- | --- | --- | --- | --- | --- | --- |
| 1 year | 480 | 0.660 | 0.619–0.701 | 2.89 | 64.20 | 61.50 |
| 3 years | 286 | 0.642 | 0.598–0.686 | 2.78 | 62.80 | 59.70 |
| 5 years | 118 | 0.623 | 0.568–0.678 | 2.70 | 61.30 | 57.90 |

Abbreviations: AUC, area under the receiver operating characteristic curve; CI, confidence interval; NLR, neutrophil-to-lymphocyte ratio; ROC, receiver operating characteristic.

Supplementary Table S10A. Performance of the multivariable logistic model

| Performance measure | Apparent estimate | Bootstrap-corrected estimate | 95% CI |
| --- | --- | --- | --- |
| AUC | 0.785 | 0.769 | 0.748–0.820 |
| Calibration intercept | 0.000 | −0.021 | −0.118–0.082 |
| Calibration slope | 1.000 | 0.921 | 0.806–1.036 |
| Brier score | 0.176 | 0.183 | 0.161–0.198 |

Abbreviations: AUC, area under the receiver operating characteristic curve; CI, confidence interval.

Supplementary Table S10B. Performance of the complementary Cox model

| Performance measure | Apparent estimate | Bootstrap-corrected estimate | 95% CI |
| --- | --- | --- | --- |
| Harrell’s C-index | 0.755 | 0.739 | 0.710–0.771 |
| 1-year time-dependent AUC | 0.777 | 0.758 | 0.720–0.798 |
| 3-year time-dependent AUC | 0.750 | 0.734 | 0.700–0.768 |
| 5-year time-dependent AUC | 0.742 | 0.721 | 0.680–0.761 |

Abbreviations: AUC, area under the receiver operating characteristic curve; CI, confidence interval; C-index, concordance index.

Supplementary Table S11. Exploratory available-case analysis of TURBT quality parameters

| TURBT quality parameter | Available data, n/N (%) | Overall cohort | Recurrence group | Non-recurrence group | P value |
| --- | --- | --- | --- | --- | --- |
| Detrusor muscle status in the initial TURBT pathology report | 558/605 (92.2) | — | 218/237 (92.0) | 340/368 (92.4) | 0.150 |
| Present, n/N (%) | — | 449/558 (80.5) | 167/218 (76.6) | 282/340 (82.9) | — |
| Absent, n/N (%) | — | 87/558 (15.6) | 42/218 (19.3) | 45/340 (13.2) | — |
| Not assessable from the available report, n/N (%) | — | 22/558 (3.9) | 9/218 (4.1) | 13/340 (3.8) | — |
| Resection technique at initial TURBT | 517/605 (85.5) | — | 199/237 (84.0) | 318/368 (86.4) | 0.180 |
| En bloc TURBT, n/N (%) | — | 112/517 (21.7) | 37/199 (18.6) | 75/318 (23.6) | — |
| Conventional TURBT, n/N (%) | — | 405/517 (78.3) | 162/199 (81.4) | 243/318 (76.4) | — |
| Imaging modality at initial TURBT | 492/605 (81.3) | — | 188/237 (79.3) | 304/368 (82.6) | 0.168 |
| Enhanced imaging used, n/N (%) | — | 91/492 (18.5) | 29/188 (15.4) | 62/304 (20.4) | — |
| └ Narrow-band imaging, n/N (%) | — | 72/492 (14.6) | 23/188 (12.2) | 49/304 (16.1) | — |
| └ Photodynamic/blue-light imaging, n/N (%) | — | 19/492 (3.9) | 6/188 (3.2) | 13/304 (4.3) | — |
| White-light imaging only, n/N (%) | — | 401/492 (81.5) | 159/188 (84.6) | 242/304 (79.6) | — |
| Repeat TURBT performed, n (%) | 605/605 (100.0) | 99 (16.4) | 49 (20.7) | 50 (13.6) | 0.021 |
| Time to repeat TURBT, days, median (IQR) | 99/99 (100.0) | 42 (35–49) | 43 (36–50) | 41 (34–48) | 0.274 |
| Residual tumor at repeat TURBT, n/N (%) | 99/99 (100.0) | 40/99 (40.4) | 25/49 (51.0) | 15/50 (30.0) | 0.033 |
| Upstaging at repeat TURBT, n/N (%) | 99/99 (100.0) | 8/99 (8.1) | 6/49 (12.2) | 2/50 (4.0) | 0.160 |

Abbreviations: IQR, interquartile range; TURBT, transurethral resection of bladder tumor.

Supplementary Figure S1. Restricted cubic spline analysis of the association between baseline NLR and recurrence hazard.

Supplementary Figure S2. Time-dependent ROC curves of baseline NLR for recurrence-free survival at 1, 3, and 5 years.

Supplementary Figure S3. Apparent and bootstrap-corrected calibration curves of the multivariable logistic model.
